# Functionalization of Polypropylene by TiO_2_ Photocatalytic Nanoparticles: On the Importance of the Surface Oxygen Plasma Treatment

**DOI:** 10.3390/nano14161372

**Published:** 2024-08-22

**Authors:** Karolina Zajac, Joanna Macyk, Konrad Szajna, Franciszek Krok, Wojciech Macyk, Andrzej Kotarba

**Affiliations:** 1 Faculty of Chemistry, Jagiellonian University in Kraków, Gronostajowa 2, 30-387 Krakow, Poland; karolina.zajac@doctoral.uj.edu.pl (K.Z.); wojciech.macyk@uj.edu.pl (W.M.); 2 InPhoCat—Innovative Photocatalytic Solutions Sp. z o. o., Brzask 49, 30-381 Krakow, Poland; jmacyk@inphocat.pl; 3 Doctoral School of Exact and Natural Sciences, Jagiellonian University, Prof. St. Łojasiewicza 11, 30-348 Kraków, Poland; 4 Marian Smoluchowski Institute of Physics, Faculty of Physics, Astronomy and Applied Computer Science, Jagiellonian University, Lojasiewicza 11, 30-348 Krakow, Poland; konrad.szajna@uj.edu.pl (K.S.); franciszek.krok@uj.edu.pl (F.K.)

**Keywords:** TiO_2_, polypropylene, surface functionalization, plasma treatment, nanocomposite, photocatalyst

## Abstract

A new two-step method for developing a nanocomposite of polypropylene (PP) decorated with photocatalytically active TiO_2_ nanoparticles (nTiO_2_) is proposed. This method involves the low-temperature plasma functionalization of polypropylene followed by the ultrasound-assisted anchoring of nTiO_2_. The nanoparticles, polymeric substrate, and resultant nanocomposite were thoroughly characterized using nanoparticle tracking analysis (NTA), microscopic observations (SEM, TEM, and EDX), spectroscopic investigations (XPS and FTIR), thermogravimetric analysis (TG/DTA), and water contact angle (WCA) measurements. The photocatalytic activity of the nanocomposites was evaluated through the degradation of methyl orange. The individual TiO_2_ nanoparticles ranged from 2 to 6 nm in size. The oxygen plasma treatment of PP generated surface functional groups (mainly -OH and -C=O), transforming the surface from hydrophobic to hydrophilic, which facilitated the efficient deposition of nTiO_2_. Optimized plasma treatment and sonochemical deposition parameters resulted in an active photocatalytic nTiO_2_/PP system, degrading 80% of the methyl orange under UVA irradiation in 200 min. The proposed approach is considered versatile for the functionalization of polymeric materials with photoactive nanoparticles and, in a broader perspective, can be utilized for the fabrication of self-cleaning surfaces.

## 1. Introduction

Polypropylene (PP), due to its vast diversity of applications, is one of the most widespread polymeric materials in the world. It gained popularity almost instantly after its discovery, and the production of PP steadily increased [1]. Diverse types of industries, including biomedical, automobile, aerospace, textiles, and packaging, to mention a few, employ polypropylene using their unique and adjustable characteristics. The most crucial advantages of these materials are chemical resistance, mechanical properties, and cost-effectiveness [2]. Polypropylene properties could be additionally modified via chemical and physical methods, among which are copolymerization and structural modification using additives and forming composites. The resultant materials could be processed in many ways, like injection or extrusion, and form final products. From the practical point of view, surface functionalization is particularly interesting, where the enhancement and/or addition of new surface properties is achieved without changing the bulk characteristics [3,4].

Currently, one of the modern trends in polymeric surface functionalization concerns antibacterial and self-cleaning surfaces, which can be achieved through the introduction of photocatalytic nanoparticles on the surface [5]. When irradiated, the photocatalysts generate reactive oxygen species such as hydroxyl radical (HO^•^) and superoxide radical (O_2_^•–^). The primarily formed O_2_^•–^ species start a cascade of ROS formation, including H_2_O_2_, which, together with HO^•^, can destroy a broad range of contaminants. They typically include inanimate matter, such as inorganic/organic substances, and living organisms, such as bacteria, fungi, and algae. Inorganic contaminants such as dust could be washed away due to superhydrophilic properties. In this case, the water contact angle should be below 10° so the water droplets can spread over and clean the surface [6]. Organic impurities could be removed from the surface via total degradation to CO_2_ and H_2_O (mineralization) by photogenerated charges or reactive oxygen species. The main mechanism in destroying bacterial cell walls also involves damage to bacteria cell walls and operates for both Gram-negative and Gram-positive strains. However, it should be noted that the generated oxygen radicals are short-lived and are not found further than 1 μm from the photocatalyst surface [7]. As the distance between the bacteria and the photocatalyst is essential for the self-disinfection process of all surfaces, the preparation of a system requires a high dispersion of photoactive nanoparticles over the polymer surface. 

Titanium dioxide in the form of nanoparticles is one of the best-known robust photocatalysts with superior properties, particularly photoactivity, nontoxicity, and commercial availability. TiO_2_-based photocatalytic systems, including films, have been shown to effectively remove organic pollutants and destroy pathogens in water, as reviewed in [8]. Thus, the deposition of titanium dioxide nanoparticles on the polymers could effectively trigger their photocatalytic cleaning [9,10]. The most commonly used approach is to add nanoparticles to monomers before forming them into a final product. There are several methods reported for the successful preparation of PP-nTiO_2_ composites like the direct blending of titania NPs with polymers, in situ formation of NPs within the polymeric matrix, copolymerization of surface-modified NPs, and grafting or self-assembly of NPs and polymers [11,12]. It is worth mentioning that in such a case, a majority of nanoparticles are located in the bulk and, due to limited light penetration, are photocatalytically inactive. The bulk embedment could also result in the undesired modifications of bulk properties of the commercial polymeric materials [13]. 

Considering the above considerations, optimal functionalization should be limited to the surface decoration of polymeric materials with photocatalytic nanoparticles. The polypropylene surface is, however, chemically inert, which is a huge advantage for its stability in harsh chemical environments. However, it also makes functionalization difficult, especially via coating or attachment of nanoparticles [14]. 

From a practical point of view, the best approach is to create surface chemical bonds between polypropylene and TiO_2_ nanoparticles. The strategy involves the generation of abundant surface functional groups on the polypropylene to enhance nanoparticle anchoring [15]. For this purpose, the effective modification of the PP surface can be achieved by plasma treatment. The exposure of polymeric surfaces to ionized gas molecules, e.g., oxygen plasma, effectively changes their properties, like wettability and adhesion. Using low-temperature plasma, the surface properties can be changed effectively by generating functional groups while preserving the bulk properties of the polymeric materials [16,17]. Moreover, the surface modification could be precisely controlled by adjusting plasma parameters (power, oxygen partial pressure, and treatment time) [18,19]. Such functionalized surfaces can then be decorated with photoactive nanoparticles with the use of a sonochemical process [20,21]. The advantages of applying ultrasound for inorganic particle deposition over polymeric surface consist in employing ultrasound energy not only for the deposition process but also for softening the polymeric matrix, thus making the nanoparticle embedding effective. Consequently, the final product is expected to exhibit higher mechanical stability [22].

In this paper, oxygen plasma treatment on polypropylene material has been investigated. New functional groups generated on the polymer surface after plasma treatment were monitored by the water contact angle, while their chemical nature was characterized by employing FTIR and XPS analysis. The process of depositing nanoparticles on the surface was performed using the sonochemical method, modifying the sonication time and concentration of the nTiO_2_ suspension. The influence of oxygen surface groups on the attachment and dispersion of the TiO_2_ nanoparticles was documented by SEM images. The photocatalytic activity was tested in the methyl orange (as model contamination compound) degradation test using UV irradiation. The effect of plasma treatment on the properties of the obtained nTiO_2_/PP composites is discussed in terms of the nanoparticle dispersion and related photocatalytic activity.

## 2. Materials and Methods

### 2.1. Material Preparation

Titanium dioxide nanoparticles (CCA 100 BS) were purchased from Cinkarna Celje d. d. (Celje, Slovenia). The TiO₂ had an anatase crystal structure with a specific surface area of approximately 250 m^2^/g. The nanoparticles were supplied in a 20% stabilized aqueous suspension with a neutral pH (7–9), which was diluted to 1% or 0.1% before the experiments.

In the experiments, 1 mm thick polypropylene sheets (Merck, Darmstadt, Germany) were used. The sheets were washed with isopropanol and dried in air to prepare the surface for further functionalization and measurement.

To functionalize the polymer’s surface, oxygen plasma treatment (FEMTO system, Diener Electronics, Ebhausen, Germany) was used with a controlled oxygen partial pressure of 0.2 mbar, plasma generator power of 100 W, and a defined sample exposure time (0, 1, 2, 5, or 10 min).

A sonochemical method was used to anchor the nanoparticles to oxygen plasma-modified polypropylene. After the plasma treatment, the polymer was immediately immersed in 5 mL of titanium dioxide solution (0.1%/0.2%/0.5%/1%). The samples were irradiated at a frequency of 20 kHz, amplitude of 30%, and time from 1 min to 5 min using a homogenizer (Ti-horn, Q500, QSonica, Newtown, CT, USA). A cold-water bath was used to avoid overheating.

### 2.2. Nanoparticle Tracking Analysis (NTA)

The hydrodynamic diameter of the nanoparticles was investigated using the nanoparticle tracking analysis method with Nanosight LM10 (A.P. Instruments, Warsaw, Poland). The instrument’s software calculates the particle diffusion coefficient and counts the particles’ hydrodynamic diameter using the Stokes–Einstein equation. Every solution was investigated three times. 

### 2.3. Attenuated Total Reflection–Fourier Transformation Infrared Spectroscopy (ATR-FTIR)

To examine changes in chemical groups on the surface after the plasma treatment, the ATR-FTIR method was used with the Nicolet Summit FTIR Spectrometer (Thermo Fisher Scientific, Waltham, MA, USA) equipment. The polypropylene samples were measured right after the plasma treatment. Each studied sample was scanned 64 times in the range of 600–4000 cm^−1^.

### 2.4. X-ray Photoelectron Spectroscopy (XPS)

To identify the surface functional groups formed during the plasma treatment, the polypropylene samples were investigated by XPS. The measurements were conducted in an ultrahigh vacuum chamber (vacuum level > 5 × 10^−9^ mbar) using an SES R4000 analyzer (Gammadata Scienta, Seattle, WA, USA) with a monochromatic Al K_α_ X-ray source (1486.6 eV) at 250 W (pass energy: 100 eV) for the survey and narrow scans. The received XPS spectra were analyzed using the Casa-XPS 2.3.15 software. The electron binding energy of the C 1*s* peak was calibrated at 285 eV.

### 2.5. SEM

The fabricated nanocomposites were investigated using an FEI (Hillsboro, OR, USA) Quanta 3D FEG field-emission scanning electron microscope (SEM) at low vacuum conditions (10 mbar of H_2_O pressure, evaporated from a distilled water reservoir) in the so-called Environmental SEM (ESEM) mode. This experimental setting was required due to the non-conductive nature of the polymeric substrate. To avoid disturbing the morphology of the nanocomposite, the samples were investigated in their native forms without any additional coatings. To verify the elemental composition of the nanocomposite, EDX measurements were also carried out for specific areas of interest.

### 2.6. TG/DTA

Thermogravimetric measurements with differential thermal analysis were performed with TGA 3+ equipment (Mettler Toledo, Warsaw, Poland). The measurements were carried out under airflow in the temperature range of 30–700 °C with a heating rate of 10 °C/min. The measured mass loss of samples (~7 mg) was normalized to 100% for the reliable comparison of the thermogravimetric profiles. 

### 2.7. Photocatalytic Tests

The photocatalytic activity was tested using the methyl orange photodegradation reaction under UV-A light (365 nm) using a Sylvania blacklight bulb 20 W (Newhaven, UK). The prepared nanocomposites—polypropylene (35 mm × 8 mm) with different nTiO_2_ coatings—were placed in a quartz cuvette with standard 3.5 mL of methyl orange aqueous solution (25 mmol/dm^3^). Absorbance was measured periodically by UV-Vis spectrometer (model 1900i, Shimadzu, Kioto, Japan) to determine changes in the methyl orange concentration upon illumination. The characteristic absorbance maximum for methyl orange at 464 nm was used for kinetic measurements.

### 2.8. Diffusion Reflectance Spectroscopy

Ultraviolet-visible diffuse reflectance spectra (UV-vis DRS) were recorded for the nanocomposites (nTiO₂/PP) using a Perkin Elmer (Waltham, MA, USA) Lambda 365 instrument equipped with an integrating sphere. The results were calculated using the Kubelka–Munk equation.

### 2.9. XRD Measurements

The crystallinity of the photocatalyst (TiO₂ powder) was examined using XRD measurements before and after the photocatalytic processes. PXRD patterns were collected using a Bruker (Billerica, MA, USA) D8 Advance Eco diffractometer equipped with a Cu sealed tube radiation source and a capillary stage. Powder samples were loaded into glass capillaries (0.5 mm in diameter). The background in the obtained diffractograms was corrected using the DIFFRAC algorithm implemented in the DIFFRAC.EVA V5 program.

## 3. Results

### 3.1. Polypropylene Substrate

**Figure 1:**
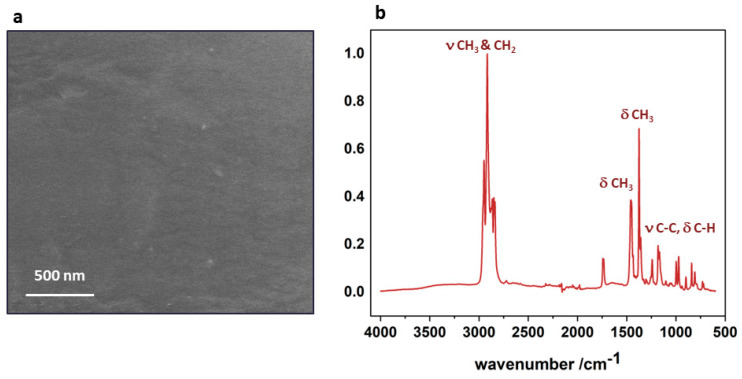
Pristine polypropylene substrate: (**a**) SEM image and (**b**) ATR-FTIR spectrum indicating the main functional groups in the polypropylene structure.

Before the preparation of the nTiO_2_/PP nanocomposite, the main constituents were characterized in their pristine forms. The SEM and FTIR characterization of the polypropylene substrate is shown in Figure 1. The SEM image demonstrates that the polypropylene exhibits a fairly smooth surface morphology on the micrometer scale, with scratches or irregularities caused by the production process not typically observed for polymers. A surface examination using ATR-FTIR (Figure 1b) provides insight into the chemical structure of the polypropylene. All the characteristic bands present in the polypropylene fingerprint can be easily observed [23], whereas no maxima characteristic for any contaminants or impurities was identified.

### 3.2. Characteristics of Titanium Dioxide Nanoparticles

**Figure 2:**
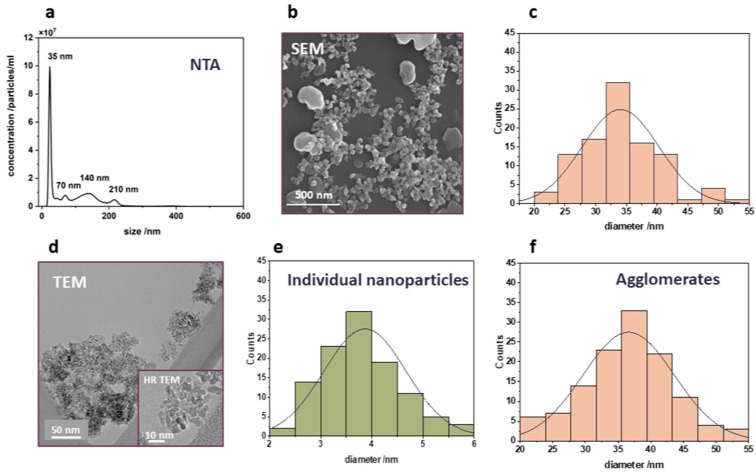
Titanium dioxide nanoparticles: (**a**) nanoparticle tracking analysis showing the hydrodynamic diameter; (**b**) SEM image of TiO_2_; (**c**) particle size distribution based on the SEM images; (**d**) TEM image showing that the observed SEM nanoparticles are agglomerates of smaller TiO_2_ crystallites, with the insert presenting a high resolution (HR-TEM) image; (**e**) size distribution of individual TiO_2_ crystallites; and (**f**) size distribution of agglomerates from TEM images.

As the photocatalytic reactions occur on the surface of the photocatalyst, the size of the photocatalyst particles is a crucial factor for the efficacy of photocatalytic processes. Therefore, the second constituent of the prepared nanocomposites—photoactive titania nanoparticles—was characterized in terms of their size. The results are presented in Figure 2a–f. The most abundant hydrodynamic diameter of the used nTiO_2_ determined with the method of nanoparticle tracking analysis was found to be 35 nm (Figure 2a). It is worth noting that other peaks at 70 nm, 140 nm, and 210 nm represent the multiplicity of the smallest particle diameter and can be attributed to agglomerates, which are formed due to a strong interaction between nanoparticles. The size distribution of the nanoparticles was also examined by SEM, showing a particle size range between 20 and 55 nm (Figure 2b). The majority of the particles present are approximately 35 nm in size, as can be inferred from the particle size distribution presented in Figure 2c. However, bigger particles of around 200 nm were also occasionally observed. The results of the EDX analysis confirmed that all the particles contain only titanium and oxygen. To analyze the nanoparticles in more detail, the TEM analysis was conducted. Figure 2d shows a typical TEM image of the TiO₂ nanoparticles, revealing that the nanoparticles observed in the SEM are actually agglomerates of much smaller crystallites. An inset in Figure 2d provides an HR-TEM image, illustrating the individual nanoparticles at higher magnification. As shown in Figure 2e, the size distribution of the individual TiO₂ crystallites ranges from 2 to 6 nm. However, when the entire agglomerates are measured based on the TEM images (Figure 2f), their size matches the 35 nm diameter observed in SEM.

### 3.3. Surface Functional Groups

**Figure 3:**
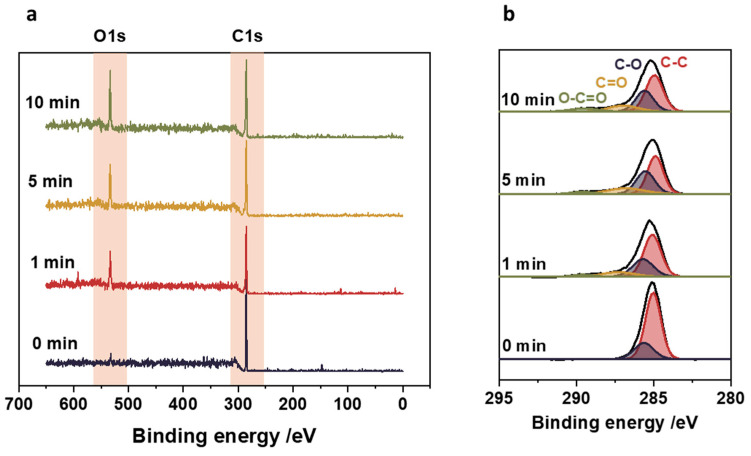
XPS results for polypropylene surface before and after oxygen plasma treatment. (**a**) XPS survey scans showing the increase in surface oxygen to carbon ratio with the plasma treatment time; (**b**) narrow XPS scan of the C 1*s* peak indicating the presence of hydroxyl (-OH) and carbonyl (C=O) groups, and the appearance of carboxyl bonds (O–C=O) upon plasma treatment.

The oxygen plasma treatment was used to increase surface adhesion and facilitate the attachment of nanoparticles to polypropylene. The main changes introduced by low-temperature oxygen plasma on the polypropylene surface consist of generating surface functional groups. Modifying the polypropylene surface depends on the chemical nature and the surface concentration of the functional groups. Therefore, the surfaces before and after the plasma treatment were characterized by XPS. The results are shown in Figure 3. The XPS survey scans show only the main constituents of the polymeric substrate: oxygen O 1*s* at 533 eV and carbon C 1*s* at 285 eV. It can be observed that before the plasma treatment, the polypropylene contained surface oxygen at a level as low as 2.8%. Its concentration, however, dramatically changes upon the plasma surface modification, as can be seen comparing the ratio of C 1*s* to O 1*s*. The longer the plasma treatment was applied, the higher the surface oxygen concentration was observed on the polypropylene, with 21.3% reached after 10 min. 

The XPS results also permit us to characterize the chemical nature of the generated functional groups. The narrow scan XPS spectra, including the analysis of C 1*s* peak, are shown in Figure 3b. As expected, a broadening of the C 1*s* peak was observed after the plasma treatment, which is related to its oxidation resulting from the generation of oxygen groups. After 1 min of oxygen plasma, the new component can be distinguished at 287 eV, corresponding to hydroxyl (-OH) and carbonyl groups (-C=O). After 5 min of the plasma treatment, a new peak at 288 eV can be distinguished and assigned to carboxyl bonds (O–C=O) [23]. Following the application of a plasma treatment for 10 min, the peak with the broadest distribution is observed, corresponding to the carbon–oxygen bonds. This clearly indicates the increase in the concentration of functional groups on the surface of the polypropylene with the time of exposure to oxygen plasma.

The surface changes induced by plasma were also confirmed by the FTIR measurements (Figure S1). After the plasma treatment, two new bands appeared in the polypropylene spectra. A broad band at 3000–3500 cm^−1^ corresponds to the vibration of –OH bonds. A second band at 1730 cm^−1^ can be assigned to –C=O vibrations [23]. Both of these maxima indicate surface oxidation. Additionally, the intensity of the observed bands increased with the time of exposure to plasma, which aligns with the XPS results described above.

**Figure 4:**
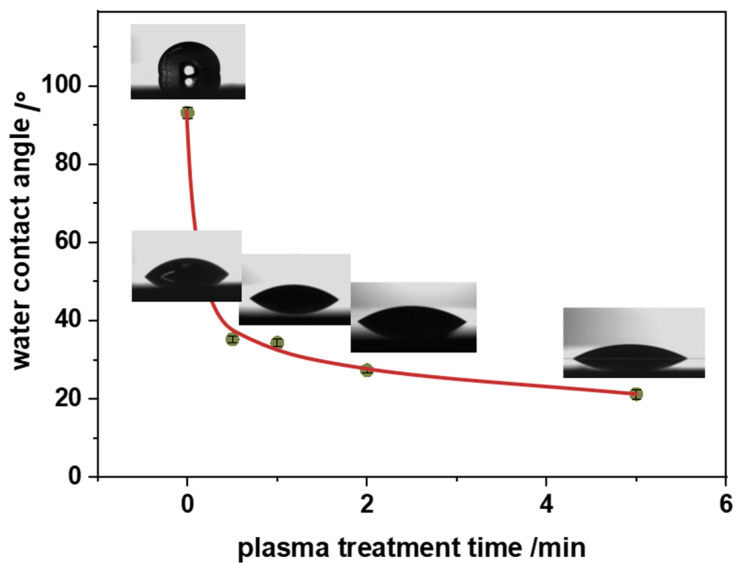
Changes in the water contact angle of the polypropylene surface as a function of oxygen plasma treatment time.

Introducing surface functional groups on the hydrophobic surface of polypropylene has very important practical implications. Since oxygen-containing functional groups are polar in nature, they stimulate stronger interactions with water molecules. Consequently, the surface becomes more hydrophilic as can be quantified experimentally by water contact angle values. They are presented as a function of the plasma treatment time in Figure 4. It can be noticed that after just 1 min of plasma, the water contact angle drops down from 95° for pristine PP to 30°. After that, a gentler water contact angle decrease is observed, reaching a stable level of about 20° after 5 min of plasma. The induced wettability of PP has a further impact on the deposition of nTiO_2_, as discussed below. 

**Figure 5:**
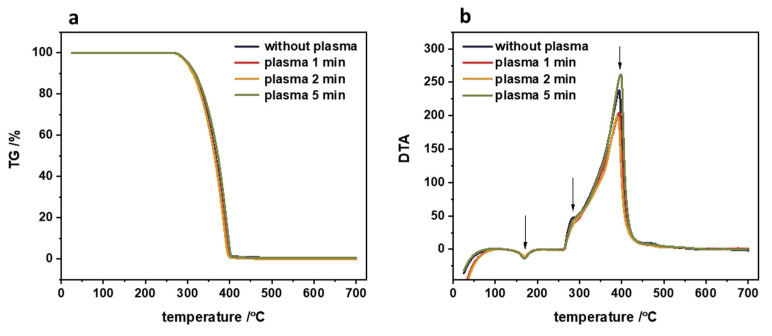
Thermogravimetric analysis of pristine polypropylene and PP after plasma treatment: (**a**) loss of mass profile and the corresponding (**b**) differential thermal analysis.

During the plasma treatment, several processes occur on the polymer surface that can be distinguished, such as cleaning, functionalization, and etching. The optimal strategy of polymer functionalization via plasma treatment is to add functional groups to the surface without disturbing the optimized bulk properties of the polymer. The effect of plasma treatment on the structural properties of the polypropylene can be easily checked by the TG/DTA measurements. The results of such thermogravimetric analysis are presented in Figure 5. The TG profiles clearly illustrated that the polymeric material is stable up to 270 °C independently of the plasma treatment time. This result strongly supports the working hypothesis that bulk polymeric properties are preserved. Additionally, there is no change in the melting temperature after the plasma treatment for 1 to 5 min (the typical melting temperature of polypropylene is approximately 170 °C [2]). Even the rapid thermal degradation of the untreated and plasma-treated polypropylene, observed in the temperature range of 270–400 °C, is the same within the experimental error.

Summarizing the effect of oxygen plasma on the polypropylene substrate, it can be concluded that while the XPS and FTIR spectroscopic results, as well as the water contact angle measurement, confirm the generation of the oxygen functional groups on the polymeric surface, its bulk properties remain unchanged. Such surface-modified polypropylene was then used as the substrate for nTiO_2_ deposition to prepare a functional nanocomposite, which is described in the next section.

### 3.4. Nanocomposite

**Figure 6:**
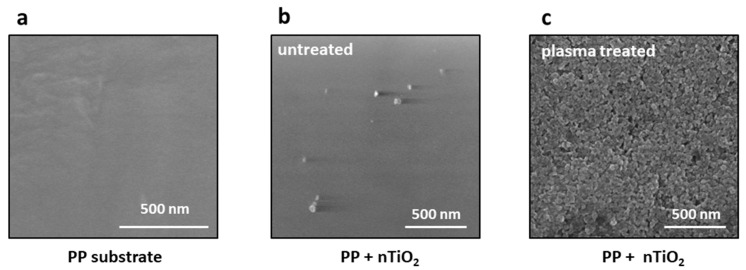
SEM images of the polypropylene substrate showing the effectiveness of TiO_2_ nanoparticle deposition on untreated and plasma-treated substrates: (**a**) pristine polypropylene surface, (**b**) TiO_2_ nanoparticles deposited on the untreated surface, and (**c**) TiO_2_ nanoparticles deposited on the plasma-treated surface.

The deposition of titania nanoparticles on the untreated or plasma-modified polypropylene substrates was performed via the sonochemical method. Figure 6 presents the SEM images comparing the effectiveness of the nTiO_2_ deposition on these surfaces together with the pristine PP substrate. The images represent the surfaces of (a) polypropylene, (b) polypropylene untreated by plasma after the deposition of nanoparticles from a 1% titanium dioxide suspension, and (c) the plasma-treated polypropylene after the deposition of nanoparticles from a 1% titanium dioxide suspension. As can be inferred from the images in Figure 6b,c, the effect of oxygen plasms is tremendous. To assure a rational comparison during the deposition process on the untreated and plasma-treated PP surfaces, the identical sonication parameters, i.e., TiO₂ concentration (1%), deposition time, power, and amplitude, were employed. Single titania nanoparticles were deposited on the untreated polypropylene substrate by oxygen plasma, whereas the plasma-treated surface exhibited a completely covered surface with TiO_2_ nanoparticles. It is evident that the efficiency of nanoparticle deposition on polymer substrates is influenced by the plasma treatment process and the introduction of functional groups on their surfaces.

**Figure 7:**
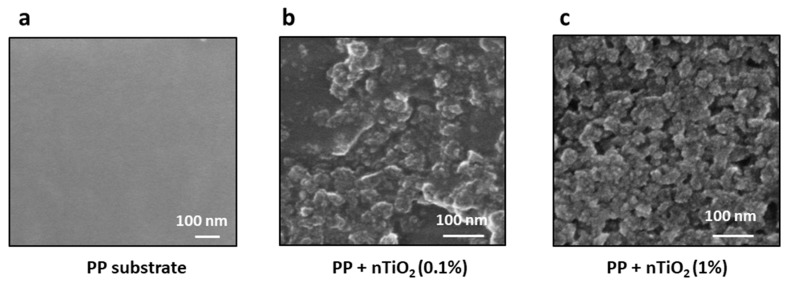
SEM images of polypropylene substrate after deposition of TiO_2_ from suspension with varying nanoparticle concentrations: (**a**) reference polypropylene substrate before nanoparticle deposition, (**b**) polypropylene surface after deposition from TiO_2_ 0.1% suspension, and (**c**) polypropylene surface after deposition from 1% TiO_2_ suspension.

In order to ascertain the optimal degree of surface coverage and, consequently, the most developed photocatalytically active surface, the effect of nanoparticle suspension concentrations on the application process was also screened. Figure 7 illustrates the scanning electron microscopy (SEM) images of a polypropylene substrate following the application of TiO₂ from 0.1% and 1% suspensions (Figure 7b, Figure 7c, respectively). Figure 7a presents a polypropylene substrate as a reference; the surface of this polymer is not decorated with nanoparticles. As expected, reduced surface coverage is evident when employing a lower initial concentration (Figure 7b). Individual nanoparticles, as well as clusters and polymer uncovered surface regions, can be distinguished. Figure 7c illustrates the full coverage of polypropylene with nanoparticles. However, the SEM image also shows pores formed between the TiO₂ nanoparticles at high coverage. Although these pores may influence the photodegradation process, it is important to note that UV light penetration will be limited in comparison to the outermost surface of the photocatalyst. The optimal concentration of TiO_2_ in the initial suspension and the most effective sonication time will yield the most favorable results. The obtained results showed that by optimizing both the plasma treatment and sonochemical deposition parameters, the surface concentration and the accessibility of the photocatalyst surface can be controlled.

### 3.5. Photocatalytic Tests

**Figure 8:**
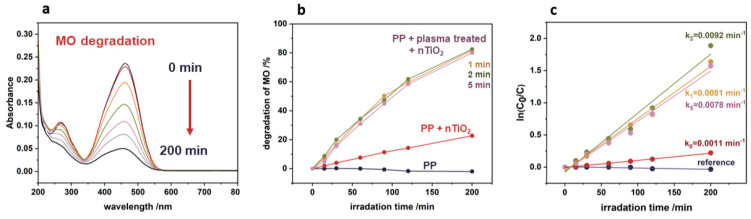
Photocatalytic activity of nTiO_2_/PP nanocomposite in methyl orange degradation under UVA irradiation: (**a**) absorbance decrease at 464 nm upon irradiation, (**b**) kinetic curves illustrating enhanced activity for plasma-treated PP composite, and (**c**) the linearized kinetic data together with the rate constants calculated based on first-order reaction kinetics.

The photocatalytic activity was investigated in the degradation of methyl orange (as a model pollutant). The degradation of methyl orange in the presence of TiO_2_/PP nanocomposite upon UVA light exposure was observed by monitoring the decrease in absorbance at the characteristic band of 464 nm in a time range of 0–200 min (Figure 8a). Based on these results, the degradation kinetics curves were determined and are presented in Figure 8b. The rate constants for the photocatalytic decomposition of methyl orange have been calculated based on the first-order reaction kinetics and the resulting values are presented beside the corresponding kinetic curves in Figure 8c. It can be observed that the polymer (PP) without nanoparticles (reference sample) does not exhibit photocatalytic activity. However, the deposition of the TiO₂ nanoparticles onto its surface strongly promotes its activity. A huge difference between the plasma-untreated (degradation of ~20% of methyl orange after 200 min) and plasma-treated PP (~80% degradation for 200 min) can also be noted. This results from the much more efficient attachment of nanoparticles onto the functionalized PP substrate (see Figure 6c). 

**Figure 9:**
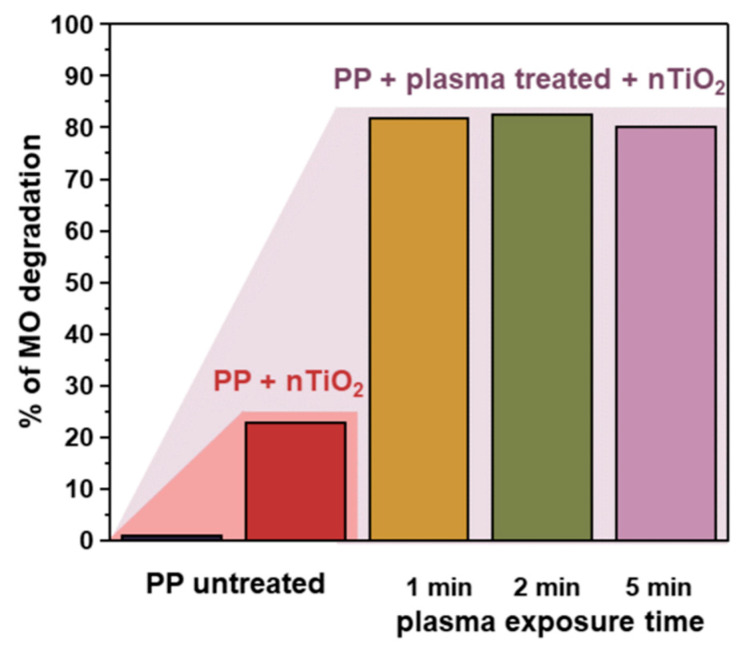
Schematic representation of the plasma treatment effect on the photocatalytic performance of the developed nTiO_2_/PP composite.

The effect of plasma treatment is schematically presented in Figure 9. It can be noted that a 1 min plasma treatment is enough to make a significant impact on the activity, and a further increase in the plasma treatment time does not result in any discernible changes To investigate the relationship between the photocatalytic activity and light absorption, UV light absorption measurements for the nanocomposites (nTiO_2_/PP) were also conducted. As shown in Figure S2 (Supplementary Information), absorption increases significantly with TiO_2_ loading. However, comparing these results with the data in Figure 9, where all the plasma-treated samples exhibit similar photocatalytic activity regardless of TiO_2_ loading, suggests that UV light absorption may not be the critical factor for the dye degradation.

**Figure 10:**
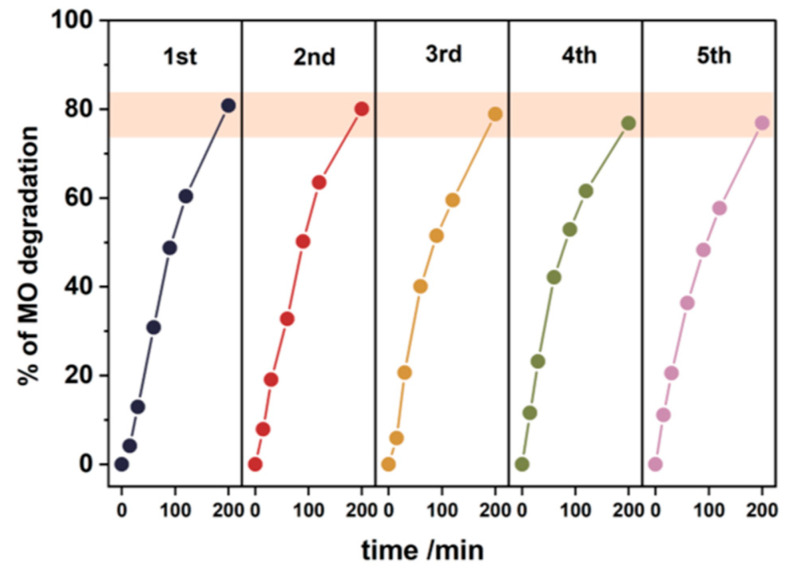
The dye degradation process over five cycles of irradiation in the presence of the nTiO₂/PP nanocomposite.

To assess the stability and photocatalytic activity of the nanocomposite systems, the dye degradation process was conducted over five cycles. The results are shown in Figure 10. In each cycle, the model pollutant was degraded by 70% to 85%. Although there is a slight decrease in the degradation of methyl orange in the subsequent cycles, it remains at a reasonably high level. This stability suggests that the nanocomposite systems maintain their photocatalytic efficiency over multiple uses. Additionally, the crystallinity of the photocatalyst (TiO₂ powder) was examined using the XRD measurements before and after the photocatalytic processes to investigate any potential structural changes. The results show that there are no visible changes in the TiO₂ structure after the UV irradiation, indicating that the nanocomposite maintains its bulk integrity under photocatalytic conditions The corresponding diffractograms can be found in the Supplementary Information in Figure S3.

**Figure 11:**
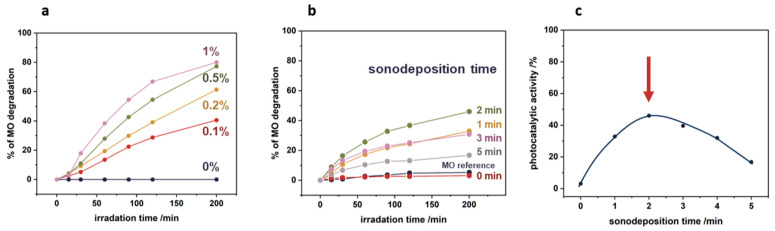
Optimization of sonochemical deposition parameters for enhanced photocatalytic activity: (**a**) effect of initial nTiO_2_ suspension concentration, (**b**) effect of sonication time on methyl orange degradation using 0.1% TiO_2_ suspension, (**c**) photocatalytic activity as a function of sonodeposition time, with 2 min identified as the optimal for maximum efficiency.

Figure 11a illustrates the effect of the initial concentration of the nTiO_2_ suspension on the degradation of methyl orange (MO) with the irradiation time. As the concentration of the TiO_2_ suspension used increases, the level of MO degradation increases in the whole-time range of irradiation. After 200 min of irradiation, for 0.1% TiO_2_ and 0.2% TiO_2_, approximately 40% and 60% degradation of the model pollutant was achieved, whereas for higher nTiO_2_ concentrations of 0.5% and 1%, the MO degradation reached 80%. As can be observed, the degradation of the model pollutant did not increase linearly with the content of TiO_2_. This may be attributed to the addition of the subsequent layers of nanoparticles, which lead to limitations in the accessibility of the TiO_2_ surface for photocatalytic processes.

To achieve optimal photocatalytic effect, the optimization of the sonochemical deposition stage was essential. The degradation results of methyl orange upon light exposure, as a function of sonication time using a 0.1% TiO_2_ suspension, are depicted in Figure 11b, while the relationship between photocatalytic activity and sonodeposition time is illustrated in Figure 11c. It becomes evident that insufficient and excessive sonication times can reduce the effective anchoring of the nanoparticles onto the polypropylene surface. The experimental data indicate that a sonication time of precisely two minutes is required to maximize the photocatalytic efficiency of the nTiO₂/PP nanocomposite system.

Furthermore, the analysis of the plasma treatment durations revealed that beyond one minute, there were no significant improvements in the photocatalytic properties of the nTiO₂/PP. This suggests that while initial plasma exposure is crucial for generating surface functional groups necessary for nanoparticle adhesion, extended treatment times do not proportionally enhance photocatalytic activity. Therefore, a balanced approach is necessary: ensuring adequate plasma treatment to functionalize the surface, coupled with optimized sonochemical deposition parameters, is essential for achieving high photocatalytic performance. The findings illustrate the importance of both surface functionalization and precise control of sonodeposition conditions in the development of effective photocatalytic nanocomposites. Finally, it is worth noting that the proposed approach combining oxygen plasma treatment and ultrasound shows significant potential and can be used for any polymer–nanoparticle system to develop functional composites for diverse specific applications.

## 4. Conclusions

The low-temperature oxygen plasma treatment effectively introduced polar surface functional groups (mainly -OH and -C=O) on the polypropylene, transforming its surface from hydrophobic to hydrophilic. This increase in wettability is evidenced by a significant reduction in the water contact angle from 95° to 20°. Moreover, the generated surface oxygen groups were found to be crucial as they enhance the interaction between the polymer and nanoparticles and thus significantly improve the anchoring efficiency of the TiO_2_ nanoparticles. The optimized parameters for the plasma treatment (100 W power, 0.2 mbar O_2_ pressure, and 1 min duration) ensured effective surface modification. The sonochemical method was found to be facile and effective for depositing the TiO_2_ nanoparticles on the plasma-treated PP surface. Optimal nanoparticle dispersion and surface coverage were achieved with a 1% TiO_2_ suspension and a sonication time of 2 min at 20 kHz, resulting in well-distributed photocatalytic TiO_2_ nanoparticles on the PP substrate. The developed nTiO_2_/PP nanocomposite demonstrated significant photocatalytic activity, degrading 80% of methyl orange under UVA irradiation within 200 min. Despite the superior surface modifications, the bulk properties of PP remained unchanged, as confirmed by the thermogravimetric analysis (TG/DTA). The polymer maintained its stability up to 270 °C and did not exhibit any changes in the melting temperature after the plasma treatment. The proposed two-step method (plasma treatment followed by sonochemical deposition) can be considered universal, versatile, and scalable, making it applicable for functionalizing various polymeric materials with photoactive nanoparticles. From a broader perspective, this approach shows high potential for designing and fabricating self-cleaning and antibacterial polymeric surfaces for diverse applications.

## Data Availability

Data are contained within the article and Supplementary Materials.

## Supplementary Materials

The following supporting information can be downloaded at: https://www.mdpi.com/article/10.3390/nano14161372/s1, Figure S1. Introducing functional groups after plasma treatment on ATR-FTIR spectra. Figure S2. Kubelka–Munk function of nTiO₂/PP nanocomposites showing material absorbance Figure S3. XRD pattern of photocatalytic phase (TiO_2_) before and after the photocatalytic processes indexing with characteristic peaks of the anatase. All authors have read and agreed to the published version of the manuscript..
