# Supplementary figures and images for: Functionalization of Polypropylene by TiO2 Photocatalytic Nanoparticles: On the Importance of the Surface Oxygen Plasma Treatment
Source: Nanomaterials (Basel). 2024 Aug 22;14(16):1372. doi: 10.3390/nano14161372 (PMC11357085; doi:10.3390/nano14161372)

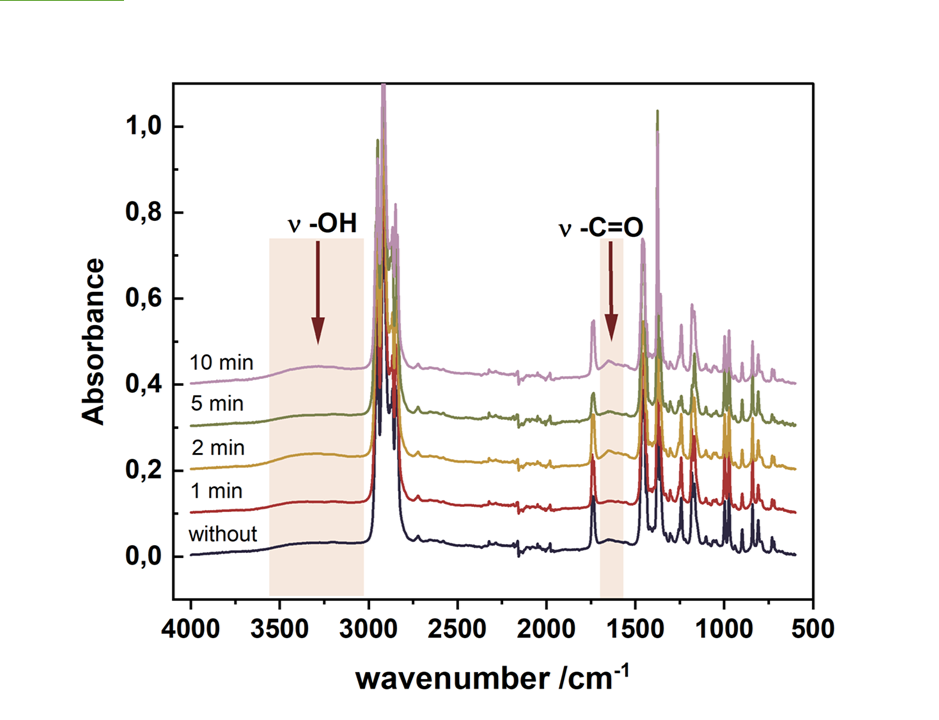

Supplement: Supplementary file 1 [file nanomaterials-14-01372-s001.zip › FigureS1.PNG]

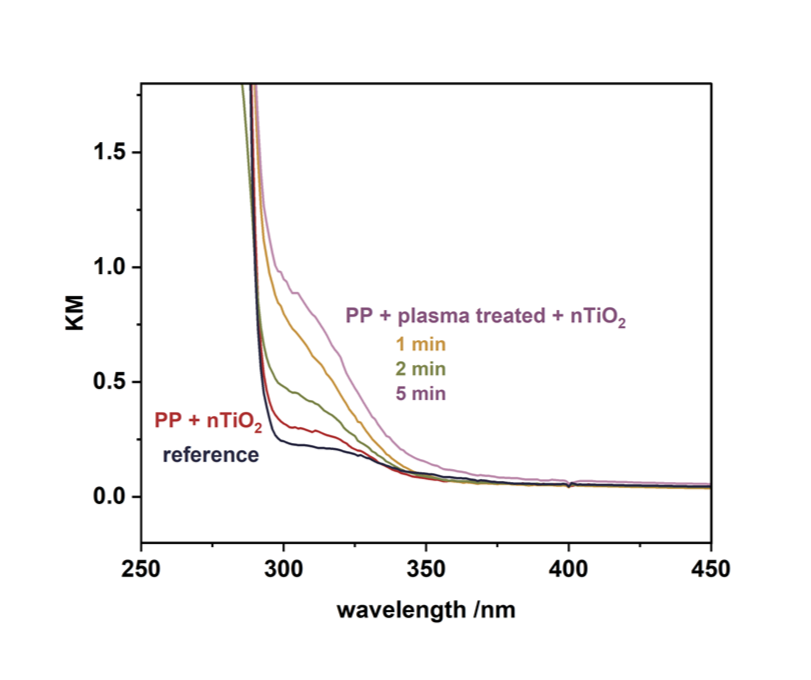

Supplement: Supplementary file 1 [file nanomaterials-14-01372-s001.zip › FigureS2.PNG]

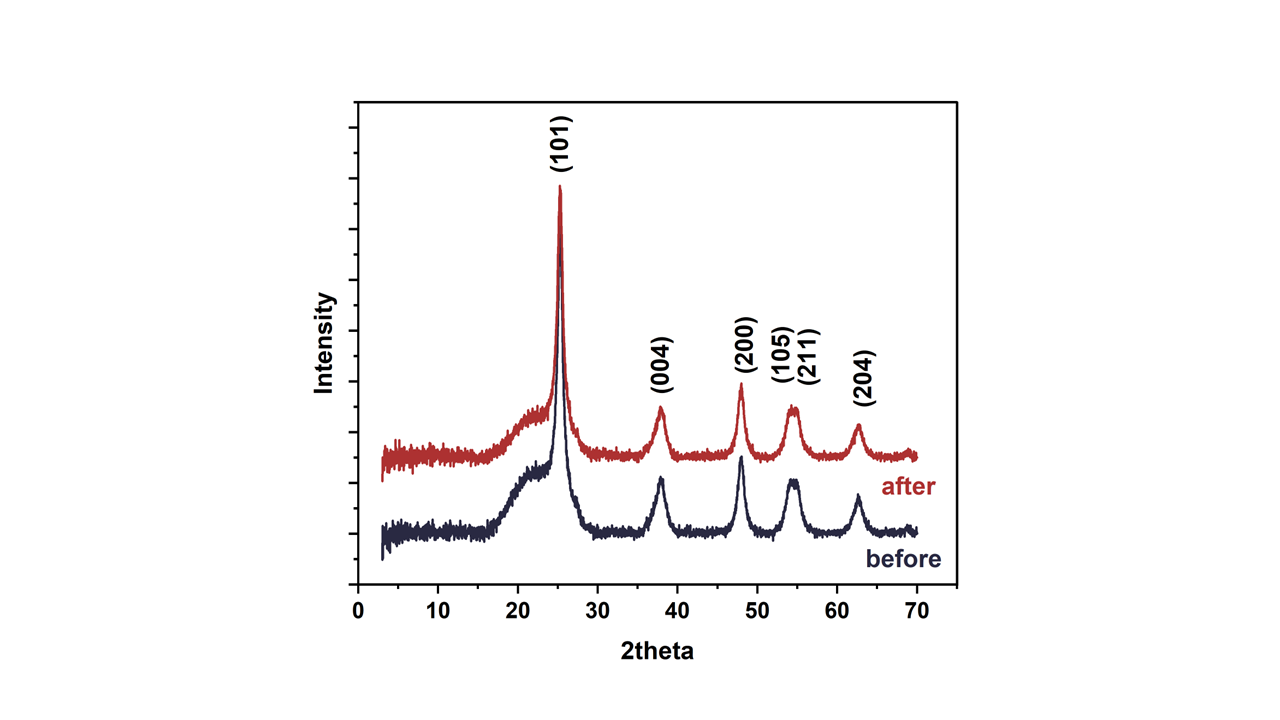

Supplement: Supplementary file 1 [file nanomaterials-14-01372-s001.zip › FigureS3.png]
